# A novel nomogram containing acute radiation esophagitis predicting radiation pneumonitis in thoracic cancer receiving radiotherapy

**DOI:** 10.1186/s12885-021-08264-y

**Published:** 2021-05-22

**Authors:** Wenjie Tang, Xiaolin Li, Haining Yu, Xiaoyang Yin, Bing Zou, Tingting Zhang, Jinlong Chen, Xindong Sun, Naifu Liu, Jinming Yu, Peng Xie

**Affiliations:** 1 Shandong First Medical University & Shandong Academy of Medical Sciences, Jinan, 250117 Shandong China; 2 Department of Radiation Oncology, Shandong Cancer Hospital and Institute, Shandong First Medical University and Shandong Academy of Medical Sciences, Jiyan Road 440, Jinan, 250117 Shandong China; 3 Department of Human Resource, Shandong Cancer Hospital and Institute, Shandong First Medical University and Shandong Academy of Medical Sciences, Jinan, 250117 Shandong China; 4 Department of Surgical Oncology, Shandong Cancer Hospital and Institute, Shandong First Medical University and Shandong Academy of Medical Sciences, Jinan, 250117 Shandong China

**Keywords:** Nomogram, Thoracic cancer, Acute radiation-induced esophagitis, Symptomatic radiation-induced pneumonitis, Radiotherapy

## Abstract

**Background:**

Radiation-induced pneumonitis (RP) is a non-negligible and sometimes life-threatening complication among patients with thoracic radiation. We initially aimed to ascertain the predictive value of acute radiation-induced esophagitis (SARE, grade ≥ 2) to symptomatic RP (SRP, grade ≥ 2) among thoracic cancer patients receiving radiotherapy. Based on that, we established a novel nomogram model to provide individualized risk assessment for SRP.

**Methods:**

Thoracic cancer patients who were treated with thoracic radiation from Jan 2018 to Jan 2019 in Shandong Cancer Hospital and Institute were enrolled prospectively. All patients were followed up during and after radiotherapy (RT) to observe the development of esophagitis as well as pneumonitis. Variables were analyzed by univariate and multivariate analysis using the logistic regression model, and a nomogram model was established to predict SRP by “R” version 3.6.0.

**Results:**

A total of 123 patients were enrolled (64 esophageal cancer, 57 lung cancer and 2 mediastinal cancer) in this study prospectively. RP grades of 0, 1, 2, 3, 4 and 5 occurred in 29, 57, 31, 0, 3 and 3 patients, respectively. SRP appeared in 37 patients (30.1%). In univariate analysis, SARE was shown to be a significant predictive factor for SRP (*P* < 0.001), with the sensitivity 91.9% and the negative predictive value 93.5%. The incidence of SRP in different grades of ARE were as follows: Grade 0–1: 6.5%; Grade 2: 36.9%; Grade 3: 80.0%; Grade 4: 100%. Besides that, the dosimetric factors considering total lung mean dose, total lung V5, V20, ipsilateral lung mean dose, ipsilateral lung V5, and mean esophagus dose were correlated with SRP (all *P* < 0.05) by univariate analysis. The incidence of SRP was significantly higher in patients whose symptoms of RP appeared early. SARE, mean esophagus dose and ipsilateral mean lung dose were still significant in multivariate analysis, and they were included to build a predictive nomogram model for SRP.

**Conclusions:**

As an early index that can reflect the tissue’s radiosensitivity visually, SARE can be used as a predictor for SRP in patients receiving thoracic radiation. And the nomogram containing SARE may be fully applied in future’s clinical work.

## Background

Radiotherapy (RT) plays a crucial role in the treatment of thoracic cancer, which carries nontrivial risks of radiation-induced lung toxicity (RILT) at the same time [1]. Moreover, this toxicity may cause a significant decline in the quality of life and the survival time. And the commonest form of RILT is radiation-induced pneumonitis (RP). It is a dose-limiting complication for thoracic cancer patients undergoing RT [2]. The lung has been reported to be sensitive to the deleterious effects of ionizing radiation [1, 3]. Acute radiation-induced pneumonitis often occurs within 6 months after finishing of RT [1, 4], which can lead to pulmonary failure and even become life-threatening [5, 6]. Besides, the radiation-induced lung injury is usually irreversible. Notably, the establishment of early RP predictors is a significant work for clinicians.

The pathogenetic process leading to RP is an integrated response to the complex organization of lung tissue, which include edema, epithelial degeneration and subsequent regeneration, invasion of alveoli by bronchial epithelium, endothelial sloughing, disruption of microvasculature, as well as atelectasis [2]. Radiation induced pulmonary damage can be varied and often long-lasting. Usually, it starts as a kind of exudative inflammation and end with scar formation which called lung fibrosis. That is the commonest end of radiation-induced lung damage [3] .

Previous studies have shown the importance of clinical characteristics, dosimetric parameters factors as well as laboratory indicators, such as pack-years, baseline pulmonary function, a history of lung resection, mean lung dose (MLD), total or ipsilateral lung volume receiving more than 2000 cGy (V20), total lung V10, total lung V13, ipsilateral lung V5, interleukin-8, recombinant human eotaxin-2, recombinant human eotaxin-22, recombinant human eotaxin-17 and so on [6–13]. In addition, genetic variants of pulmonary surfactant-associated glycoprotein D, homeodomain interacting protein kinase 2 and interleukin-4 were also reported to be associated with RP development [14–16]. And the combination of chemotherapy has also been implicated in increasing chances of developing RP [10, 17, 18].

Although numbers of predictors have been reported to be related with RP, few of them can be truly applied clinically. There is still a lack of consensus on reliable predictors for clinicians. More so, an accurate RP predictive model with superior clinical utility is urgently needed. By a long term of observation, we observed a novel and interesting phenomenon that patients with symptomatic radiation-induced pneumonitis (grade ≥ 2, SRP) often suffered severe acute radiation-induced esophagitis (grade ≥ 2, SARE) during RT. This study aimed for exploring the predictive value of SARE to SRP, and establishing a visually nomogram model for SRP among patients undergoing thoracic RT.

## Methods

### Patients cohort

From Jan 2018 to Jan 2019, patients with thoracic malignancies such as lung cancer, esophageal cancer and some mediastinal malignant tumors were included in this study prospectively. All patients were treated with thoracic RT with or without concurrent chemotherapy. In order to explore the relation between pneumonitis and esophagitis, we selected patients whose esophagus and lung tissue were both exposed to radiation. As a result, most of the included patients were central bronchogenic carcinoma and esophagus cancer with mediastinal lymph node metastasis. The type of RT was determined by both the patient and the clinician. Most of them tolerated a total dose≥5000 cGy. The inclusion criteria contained: (1) Receipt of thoracic radiotherapy; (2) Karnofsky Performance Status≥70; (3) Age ≥ 18; (4) Ipsilateral lung volume receiving 500 cGy (V5) ≥ 20% and maximum esophagus dose≥4500 cGy (in order to select patients whose esophagus and lung tissue were both exposed to radiation). The exclusion criteria included: (1) Previous history of thoracic RT; (2) Receipt of stereotactic body RT; (3) Patients with a life expectancy of less than 6 months (because they might not benefit from local radiation and might not be assessable for late lung toxicity); (4) Severe complications such as coronary heart disease, ≥grad 3 hypertension and ≥ grad 3 chronic obstructive pulmonary diseases; The fasting blood-glucose of patients with diabetes need to be controlled≤7 mmol/L. (5) Patients that didn’t come back to the hospital to take a chest computed tomography (CT) reexamination among the 6 months after RT; (6) Patients that failed to be contacted. No restrictions were placed on either the use of cytotoxic chemotherapy or the stage of disease. We seek for appropriate patients clinically, then observe and record the time, RTOG grade and duration of esophagitis symptoms during radiotherapy. All patients were followed up every month in the first 6 months after RT until the RP occurred.

### Feature definition and endpoints

We analyzed 31 continuous and categorical variables in this study. The continuous features were age, smoking pack-years, total radiation dose, number of radiation fractions, radiation fraction size, some indicators of pulmonary function (forced vital capacity FVC, forced expiratory volume in the first second FEV1, peak expiratory flow PEF, etc.), and some peripheral blood indicators before RT (erythrocyte count, white blood cell count, neutrophil count and lymphocyte count). We also collected the lowest hematology index during RT (white blood cell count, neutrophil count and lymphocyte count). We obtained some dosimetric parameters from dose-volume histogram (DVH) such as total lung V5, ipsilateral lung V20, mean esophagus dose, etc. For esophageal carcinoma or mediastinal tumor, ipsilateral lung refers to the side that received more radiation dose in RT. The time that pneumonitis symptoms appear after RT were included. The categorical features were gender, smoking status, SARE, esophagitis duration time after RT, etc. All data, including clinical parameters and the grade of RP/ARE were collected prospectively. ARE was graded in accordance with the Radiation Therapy Oncology Group (RTOG) acute radiation injury grading criteria. The primary endpoint of this study was SRP, defined in the Common Terminology Criteria for Adverse Events (CTCAE) version 5.0 [19] published by the National Cancer Institute (Table 1). The choice of RT pattern was jointly formulated by the clinicians, physicists as well as patients themselves, and the pulmonary function test was determined by the individual condition of every patient. The assessment of RP and SARE was diagnosed by 2 radiation oncologists or pulmonologists based on patients’ clinical symptoms and the range of radiographic infiltration within the radiation field during the first 12 months after radiation.

**Table 1:** The assessment criteria of RP and ARE in this research

Grade	Radiation Therapy Oncology Group grading for ARE	Common Terminology Criteria for Adverse Events (CTCAE) v5.0 for RP
0	None	None
1	Mild dysphagia or odynophagia may require topical anesthetic or non-narcotic analgesics/may require soft diet	Asymptomatic; clinical or diagnostic observations only; intervention not indicated
2	Moderate dysphagia or odynophagia may require narcotic analgesics/may require puree or liquid diet	Symptomatic; medical intervention indicated; limiting instrumental ADL
3	Severe dysphagia or odynophagia with dehydration or weight loss > 15% from pretreatment baseline/requiring N-G feeding tube, iv. fluids or hyperalimentation	Severe symptoms; limiting self-care ADL; oxygen indicated
4	Complete obstruction, ulceration, perforation, fistula	Life-threatening respiratory compromise; urgent intervention indicated (e.g., tracheotomy or intubation)
5	/	Death

Abbreviations: *iv* Injection of Vein, *ADL* activities of daily living

### Statistics

Univariate and multivariate logistic regression analysis were performed to explore features associated with SRP. We also used the Gamma rank correlation coefficient to further confirm the relation between SARE and SRP. All statistical tests were two-tailed, with a *P* value less than 0.05 considered to be statistically significant. Receiver operating characteristic (ROC) curve analysis was used to confirm optimal cut points for variables identified as influencing RP in this analysis. The variables were subjected to multivariate analysis only when a significant difference (*P* < 0.05) was computed in the univariate analysis. Cumulative incidence of SRP was calculated from the end of RT to the date of SRP occurrence or to the date of last follow-up, patients lost to follow-up were censored. All statistical analyses were performed using the SPSS version 25.0. And the nomogram model was established by the package of rms in “R” version 3.6.0.

## Results

### Patients’ characteristics

**Fig. 1:**
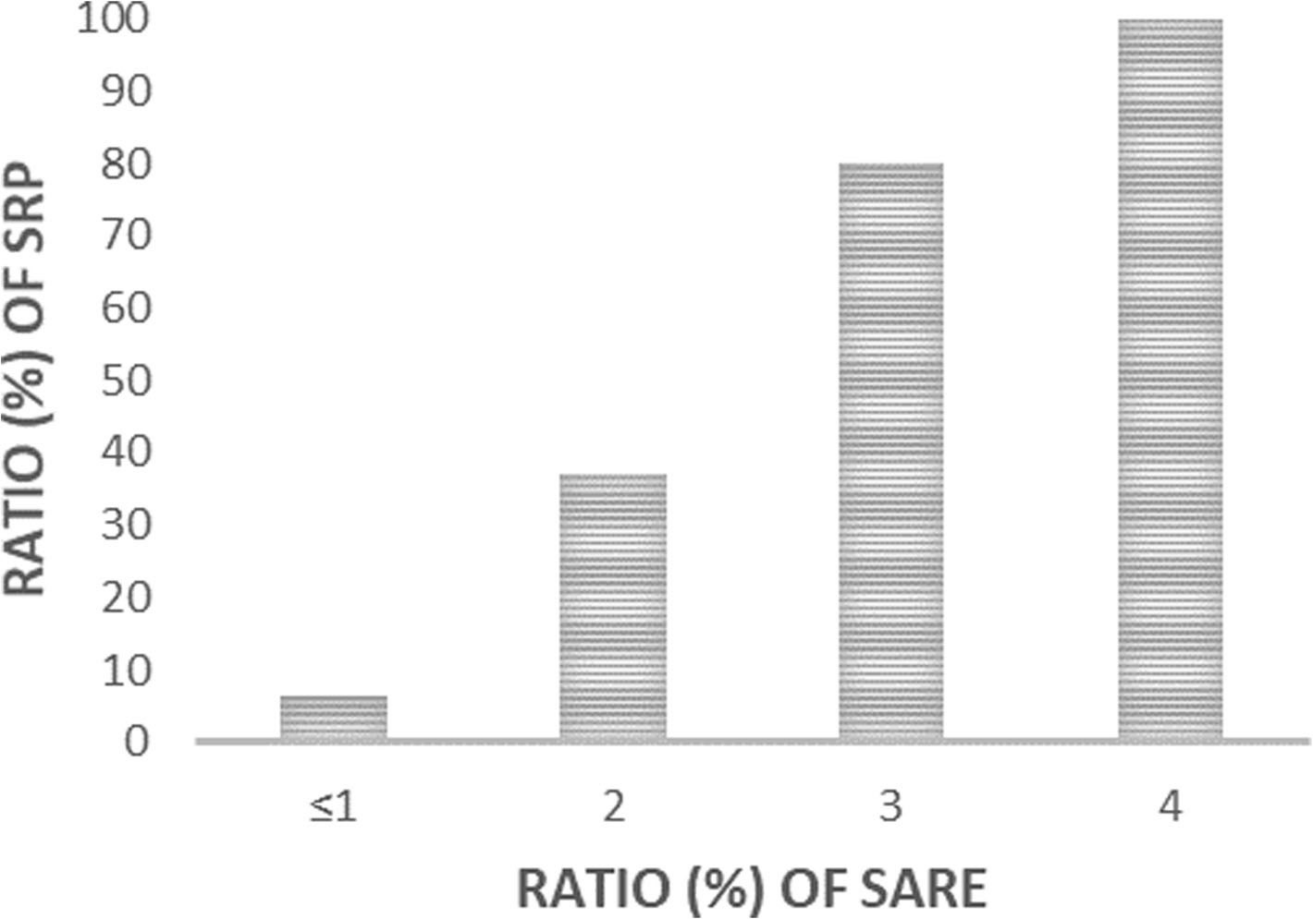
Incidence of SRP in different grades of ARE population. Abbreviations: ARE: acute radiation-induced esophagitis; SRP: severe radiation-induced pneumonitis

From Jan 2018 to Jan 2019, a total of 123 patients receiving thoracic RT were included prospectively in this study, which contained 57 (46.3%) lung cancer patients, 64 (52.0%) esophageal cancer patients and 2 (1.6%) mediastinal malignant fibrous adenoma patients. The median age was 64 (range 25 ~ 81) years old, and 94 (76.4%) patients were male. Most patients (103, 83.7%) received intensity-modulated radiation therapy (IMRT) in the study, 15 (12.2%) patients received 3D-conformal RT (3D-CRT), and 5 (4.1%) patients received tomotherapy (TOMO). The median dose of RT was 6000 cGy, which ranged from 4100 cGy to 6600 cGy. ARE appeared in this cohort were as follows: Grade 0 ~ 1 in 46 (37.4%) patients, Grade 2 ~ 5 in 77 (62.6%) patients. At the end of the follow up (July 6, 2019), 37 patients suffered SRP, 3 of them (8.1%) died of RT-related respiratory failure. And among those patients, 34 (91.9%) patients developed SARE. Table 2 shows the details of this study and patients’ basal characteristics. And the incidence of SRP in different grades of ARE is explained in Fig. 1.

**Table 2:** Patient characteristics (*N*=123)

Variables	Cohort, No. (%)
Median age	64 (range 25~81)
Sex	
Male	94(76.40)
Female	29(23.60)
Smoking status	
No smoking	52(42.30)
Current	31(25.20)
Former	40(32.50)
Tumor type	
Lung cancer	57(46.30)
Esophageal cancer	64(52.00)
Mediastinal malignant fibrous adenoma	2 (1.60)
RT modality	
IMRT	103(83.74)
3D-CRT	15(12.20)
TOMO	5 (4.06)
Total prescribed dose (cGy)	
≥6000	64(52.03)
5000~6000	24(19.51)
≤5000	35(28.46)
ARE	
Grade 0-1	46(37.40)
Grade 2-5	77(62.60)
RP	
Grade 0-1	86(69.90)
Grade 2-5	37(30.10)

Abbreviations: *OR* odds ratio, *CI* confidence interval, *RT* radiotherapy, *IMRT* intensity-modulated radiotherapy. *3D-CRT* 3D-conformal radiotherapy, *TOMO* tomotherapy; *ARE* acute radiation-induced esophagitis, *RP* radiation-induced pneumonitis

### Univariate analysis

**Fig. 2:**
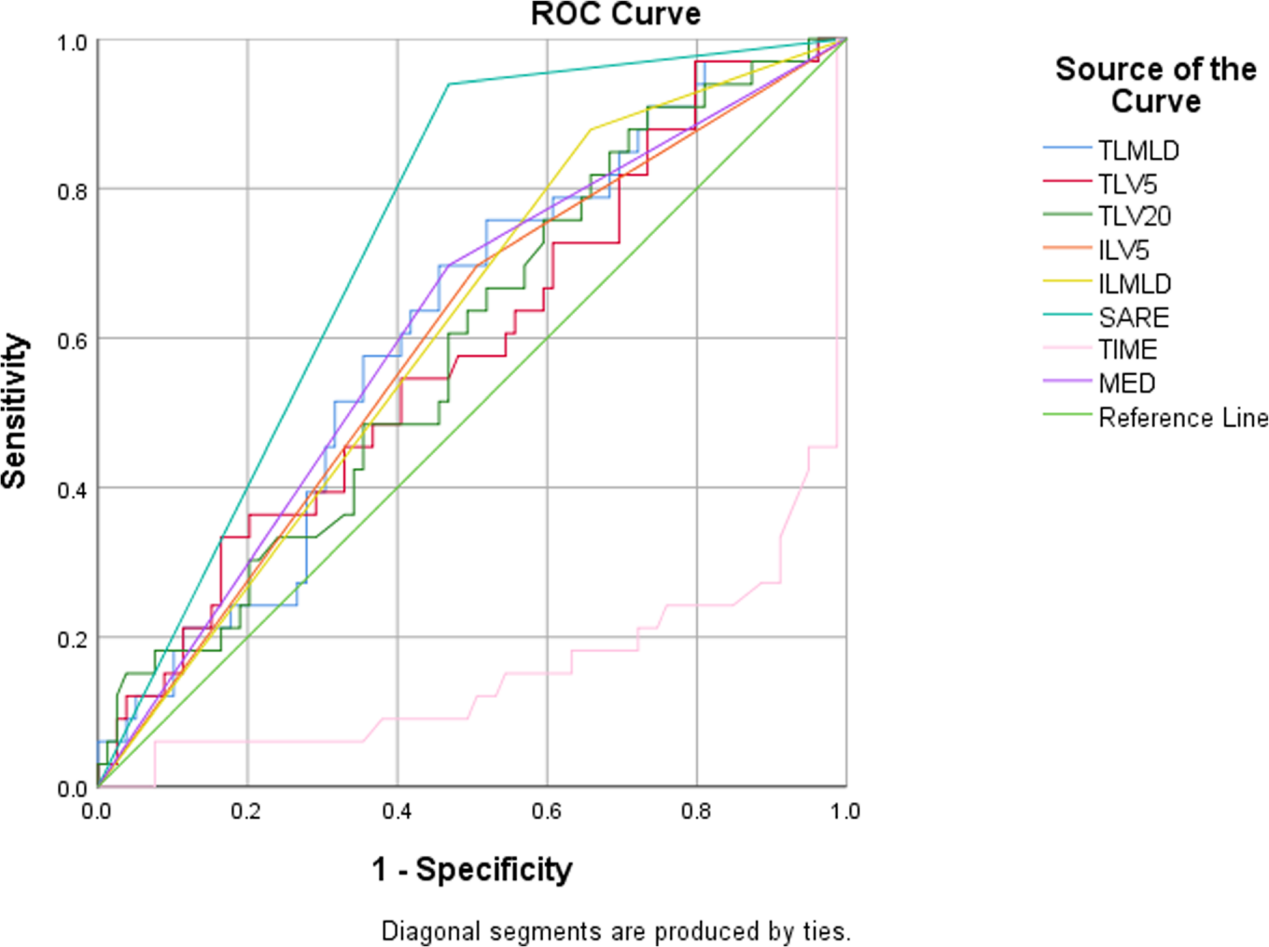
Receiver operating characteristic (ROC) curves illustrating the relative predict power of these factors. And SARE seems to be a better predictor for symptomatic radiation-induced pneumonitis (SRP) with areas under the curve (AUC) values of 0.778, *P* <0.001. Besides that, MED, AUC=0.641 *P*=0.019; IL-MLD: AUC=0.610 *P*=0.067; Abbreviations: SARE: severe acute radiation-induced esophagitis; TLMLD:total mean lung dose; ILMLD: ipsilateral mean lung dose; MED: mean esophagus dose; TIME: the time that pneumonia symptoms appear

In univariate logistic regression analysis, SARE was proved to be strongly correlated with SRP (OR 11.333, 95%CI 3.235–39.710, *P* < 0.001). Furthermore, we explored the correlation between SARE and SRP by using the Gamma rank correlation coefficient (0.838, *P* < 0.001). The sensitivity, specificity, positive predictive value and negative predictive value of SARE to predict SRP were 91.9, 50.0, 44.2 and 93.5%, respectively. The false negative rate was 8.1%. The dosimetric factors considered total lung MLD (TLMLD, OR 1.001 *P* = 0.024), ipsilateral lung MLD (ILMLD, OR 3.955, *P* = 0.018), total Lung V5 (TLV5, OR 1.034, *P* = 0.035), V20 (TLV20, OR 1.063, *P* = 0.035), ipsilateral lung V5 (ILV5, OR 2.517, *P* = 0.034), mean esophageal dose (MED, OR 1.000, *P* = 0.012) correlated with SRP. Among these results, mean ipsilateral lung dose and ipsilateral lung V5 were nonsignificant when they expressed as continuous factors, then we used ROC analysis to determine the best cutoff points for mean ipsilateral lung dose (1186.78 cGy) and ipsilateral lung V5 (55.65%). The areas under the curve (AUC) were 0.583 and 0.589, respectively. And then, we found that when patients performed as mean ipsilateral lung dose≥1186.78 cGy and ipsilateral lung V5 ≥ 55.65%, they might suffer a higher risk of RP. Besides that, the time that RP appeared after RT were also associated with the SRP (OR 0.986, *P* = 0.001). No significant correlations were found for the peripheral blood information or pulmonary function index. Table 3 summarizes these above results. And the ROC curves illustrate the predictive value of SARE and other factors (Fig. 2).

**Table 3:** Analysis of factors associated with Grade ≥2 RP

Variables	OR	Univariate *P*-value	analysis 95% CI	AUC	OR	Multivariate *P*-value	analysis 95% CI
SARE	11.333	0.000	3.235-39.710	0.778	16.763	0.000	3.638-77.230
TLMLD	1.001	0.024	1.000-1.002	0.615			
TLV5	1.034	0.035	1.002-1.067	0.586			
TLV20	1.063	0.035	1.004-1.124	0.590			
ILMLD (>1186.78 cGy)	3.955	0.018	1.270-12.317	0.610	3.557	0.048	1.013-12.495
ILV5 (>55.65%)	2.517	0.034	1.073-5.905	0.595			
MED	1.000	0.012	1.000-1.001	0.641	1.000	0.044	1.000-1.001
TIME	0.986	0.001	0.978-0.994	0.844			

Abbreviations: *OR* odds ratio, *CI* confidence interval, *SARE* severe acute radiation-induced esophagitis, *TL* total lung, *IL* ipsilateral lung, *MLD* mean lung dose, *MED* mean esophagus dose, *Time* the time that pneumonia symptoms appear, *AUC* areas under the curve

### Multivariate analysis

All above significant factors were included in the multivariate logistic regression analysis to find the most meaningful early predictors for SRP. And the multivariable logistic prediction model included three clinical variables (mean esophagus dose, mean ipsilateral lung dose and SARE), as shown in Table 3. SARE was shown to be the best independent risk predictor for the development of SRP (OR 16.763, 95%CI 3.638–77.230, *P* < 0.001).

### Nomogram model for SRP

**Fig. 3:**
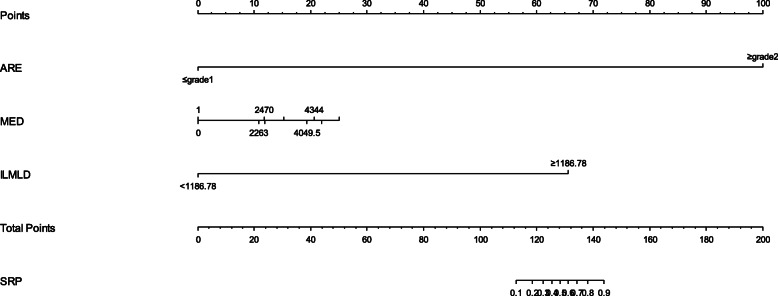
Nomogram for the prediction of SRP, based on multivariable model. Instructions: To use the nomogram, an individual patient’s value is located on each variable axis, and a line is drawn upward to determine the number of points received for each variable value. The sum of these numbers is located on the Total Points axis, and a line is drawn downward to the axes to determine the likelihood of SRP. Abbreviations: ARE: acute radiation-induced esophagitis; MED: mean esophagus dose; ILMLD:mean ipsilateral lung dose; SRP: severe radiation-induced pneumonia

**Fig. 4:**
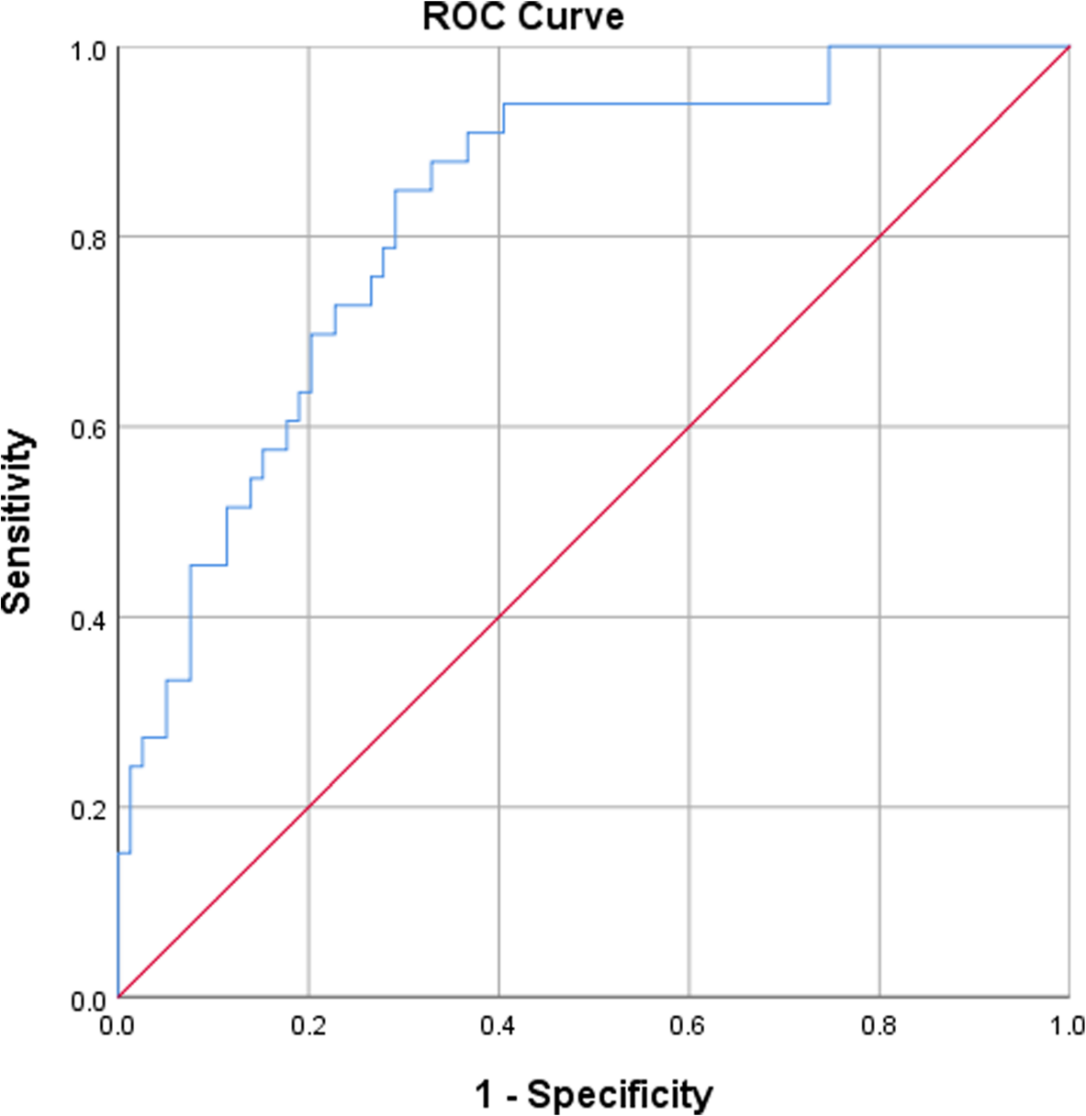
ROC curve of the nomogram model for SRP: the area under the curve is 0.827 (95% CI: 0.746-0.908, *P* <0.001)

**Fig. 5:**
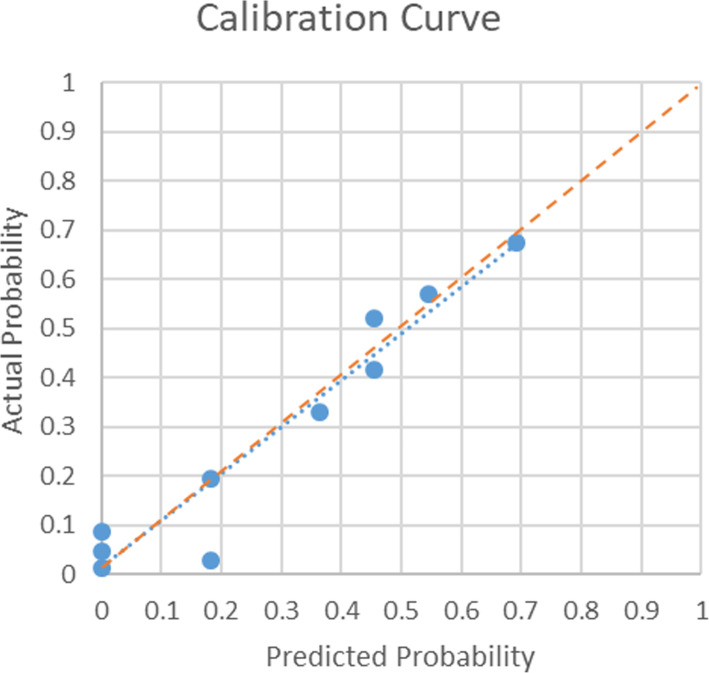
Calibration Curve. Hosmer and Lemeshow Test: Chi-square=10.975 *P*=0.203 The predicted probability is almost the same as the actual probability, and the prediction error of the model is acceptable

Finally, a visually predictive nomogram (Fig. 3) was formulated based on the results of multivariate logistic regression analysis. Fig. 4 demonstrates the area under the curve (AUC = 0.827, *P* < 0.001) of the data, and the predictive ability was estimated by AUC of ROC graphs. The ROC curve provides a visual representation of the sensitivity and specificity of measured parameters relative to RP risk. Bootstraps with 1000 resample were used for the validation of nomogram and calibration curve construction, and we can see a great match of the actual probability with the predicted probability in Fig. 5.

## Discussion

Due to the significant morbidity and potential for mortality associated with RP, it has always been a tough nut to crack for the oncologists. As described above, previous researches have made great efforts to establish reliable predictors to guide clinicians in mitigating the radiation-induced lung toxicity. And the DVH parameters of the lung has been wildly applied in clinic, such as MLD and lung V20 [7–12]. However, these factors can’t reflect the difference between individuals. In this study, we studied 31 parameters from 123 thoracic cancer patients enrolled prospectively. The results of this study demonstrated SARE, ILMLD (> 1186.78 cGy), MED were associated with SRP. And we further established an original nomogram model for symptomatic RP based on that.

To the best of our knowledge, it is the first time that SARE acts as a predictor for ≥grade 2 RP. As we all know, both the radiation-induced pneumonitis and radiation-induced esophagitis are typical radiation toxicities among thoracic RT. However, no research has linked the two together before that. The symptoms of SARE include retrosternal pain, dysphagia and odynophagia. It is easy for an experienced radiation oncologist to estimate the severity of ARE by the patient’s symptoms and physical signs during RT. SRP means that patients not only have changes on CT, but also have manifestations, such as cough, expectoration, dyspnea, etc. The assessment criteria of RP and ARE is explained in Table 1 in detail. By a long term of observation in clinic, we found a possible relation between these two inflammations and then designed a real-world study to explore the predictive value of SARE to SRP. Prior studies have explored numerous predictive factors for RP. Based on these results, we collected some of the clinical, laboratory and dosimetric parameters that we can obtain as much as possible. In order to better observe and record the initiation and development of patients’ symptoms, we chose to collect information prospectively. The results highlighted the predictive value of SARE to SRP. And we think SARE may function as an easier and earlier signal for SRP in future’s clinical work.

**Fig. 6:**
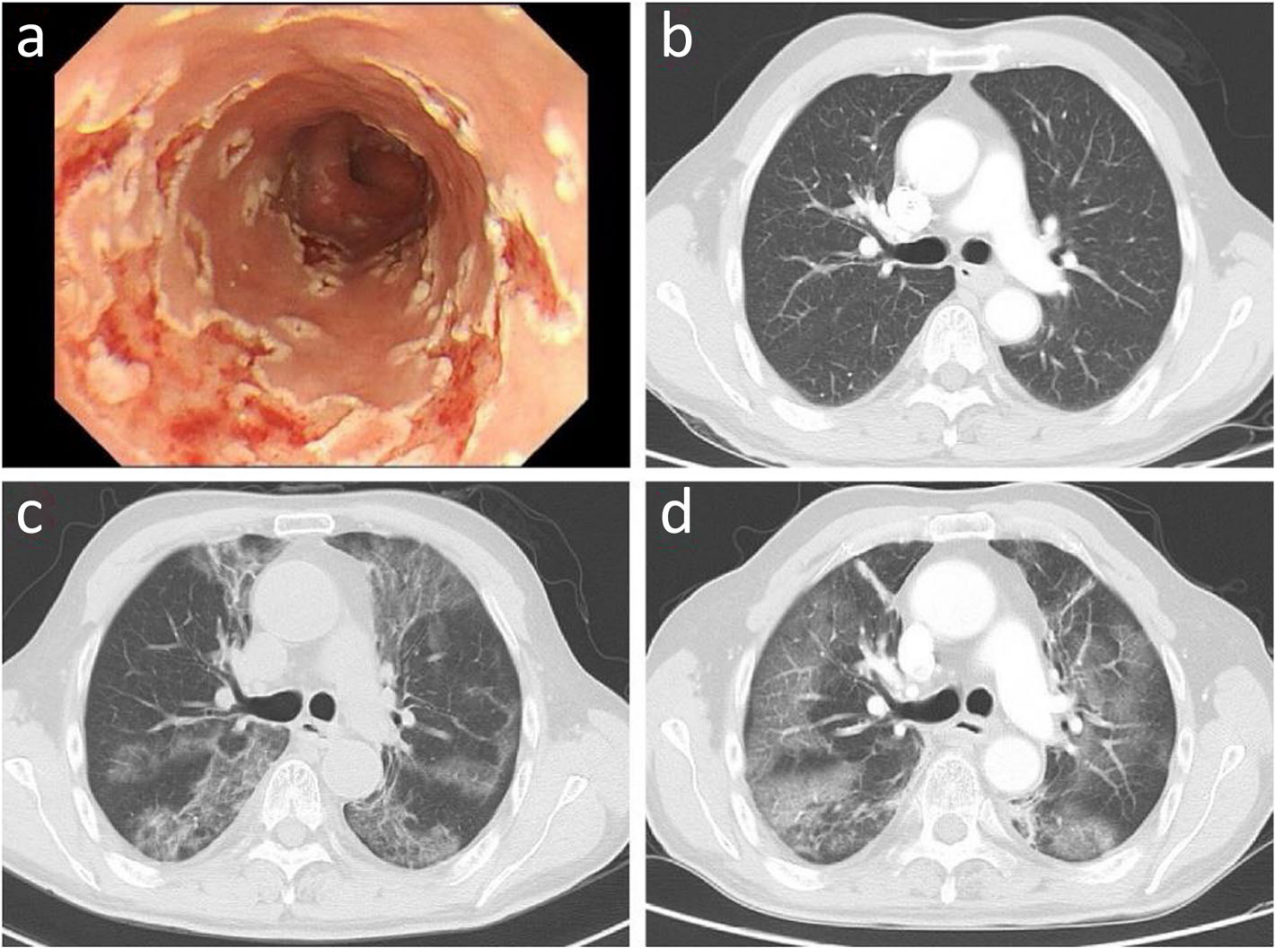
This 64-year-old esophageal cancer patient suffered grade 4 severe acute radiation-induced esophagitis (SARE) after only 10 fractions of radiotherapy. And he developed symptomatic radiation-induced pneumonitis (SRP) only 4 days after radiotherapy (RT) and then got infected of pneumocystis carinii during RT treatment. **a**: Endoscopy reveals diffuse mucosal erosion, sloughing, ulceration, and hemorrhage, and the endoscopic secretion culture revealed the fungal infection. **b**: Computed tomography (CT) image before radiotherapy; **c**: CT image that 6 days after radiotherapy; **d**: CT image that got infected of pneumocystis carinii during RP treatment.

The high incidence of SRP was estimated to be at the range of 15 ~ 40% among patients with thoracic RT by previous studies [20], and the incidence in our study (30.8%) was consistent with it. The mortality associated with RP was reported to be less than 2% [21], which was 1.6% in our study. The lethal ratio of RP was not extremely high, however, it did decrease the quality of life and led to poor prognosis [22, 23]. Moreover, the lung tissue may become more vulnerable to virus or bacterial infection. In this study, there was a 64-year-old esophageal cancer patient who died of RP related pulmonary infection. After only 10 fractions of RT, the old man suffered intractable grade 4 ARE. He felt extremely pain when swallowing and had difficulty in eating. The symptoms become worse and worse, then he can only drink a little water in the final stage of radiotherapy. The esophagoscopy found diffuse esophagus mucosal erosion, ulceration, and hemorrhage (Fig. 6a). Moreover, the symptoms continued for the subsequent treatment and even after RT. Then the pneumonitis symptoms appeared only 4 days after RT. Unfortunately, the man got infected with pneumocystis carinii during the following treatment of RP (Fig. 6b ~ d) and died soon. This case reminds us that RP can not only reduce lung tolerance but increase the chance of severe pulmonary infection. If the doctor had realized the strong connection between SARE and SRP, he/she might modulate the radiotherapy plan timely. And the subsequent tragedy may be avoided by that.

In this real-word study, we set up both the esophagus and lung dose limits to the enrollment criteria to select those patients whose lung and esophagus tissue were both exposed to radiation. In another word, these patients have a highly likelihood of both two types of inflammation. The acute esophagus toxicity is regard as the response to the radiation exposure of esophageal mucosa. Patients often develop dysphagia and odynophagia, which may lead to appetite loss and weight loss [24, 25]. These symptoms usually appear at 2 to 4 weeks during RT, and some even occur in the first 2 weeks. In addition to reducing the quality of life, SARE may cause treatment break, which is related to inferior survival results [26, 27]. There is no specific cure for it, usually, ARE acts as a kind of self-limited complication. Symptoms can disappear in 2 to 4 weeks after the completion of RT gradually [27]. Our results showed SARE was associated with two esophagus-related dosimetric indicators, they were esophagus volume receiving more than 3000 cGy (V30) and the maximum esophagus dose. These two indicators showed a weak connection with SARE (OR: 1.000 and 1.010), which couldn’t influence SRP. Thus, SARE could be used to reflect the sensitivity of individual’s normal tissue towards the radiation directly. Patients whose radiation fields contain both the lungs and esophagus need to be more vigilant about the occurrence of SARE. If someone has developed SARE, great caution should be exercised on RP prevention. He/she should pay more attention to keep warm during and after RT to avoid catching cold. And the patient should take timely chest CT to determine the lung condition and change the patient’s treatment plan if necessary.

Numerous lung related DVH parameters for RP have been widely verified in previous studies, such as total or ipsilateral lung V5, V10, V13 and V20 [6–14]. Furthermore, the dosimetric parameters of esophagus, which may have not been served as predictors for RP, were also considered in this study. And we set enrollment limits on ILV5 and MED to reduce the bias of individual dose difference among the lung and esophagus, in order to avoid their affection on RP. We collected the parameters of mean esophagus dose and esophagus V30. We found that the MED made sense both in the univariate analysis and multivariate analysis. We admitted its value as a significant predictor for SRP, but that didn’t mean it was one of the causes of RP. It served as an indirect indicator that could reflect the distribution and volume of pulmonary disease surrounding the esophagus, which eventually would be reflected in the pulmonary dosimetric outcomes. And previous study had already found that esophagus dosimetric indices were positively correlated with lung dosimetric indices [7].

There are several possible reasons that may explain the predictive value of SARE to SRP. First of all, SARE can reflect the high esophageal radiation dose [27, 28], which further implicates the increase of radiation dose in the surrounding lung tissue. Secondly, the esophagus and lung are radiosensitive organs, and the early occurrence of SARE may further indicates the high radiosensitivity of the body tissue. More than this, Previous studies have confirmed multiple inflammatory cytokines were closely related to SARE as well as SRP [10, 27, 29, 30], so it may be a signal of activation of the body’s systemic inflammatory system. However, more basic experiments are needed to better explain the relation.

Although we have obtained a considerable relation between SARE and SRP from 123 patients, it is far away to get a convincing conclusion. The main limitation of this study is the relatively small number of patients. More so, we validated the nomogram model internally by Bootstraps with 1000 resample instead of an external database. In addition, some more fundamental research toward the common nature of these two inflammations are needed to finally prove it.

## Conclusion

A possible relation between SARE and SRP was demonstrated for the first time, especially for patients with central bronchogenic carcinoma and esophageal cancer. Compared with previously reported predictors, SARE can reflect the tissue’ radiosensitivity visually. And SARE is easy to get assessed and further applied in clinic. The nomogram model containing SARE may assist the clinicians in identifying patients at great risk of SRP and guiding personalized radiation dose prescription or surveillance decisions. We hope this relation can be further confirmed by validation studies in the future.

## Data Availability

The datasets used or analyzed during the current study are available from the corresponding author on reasonable request.

## Abbreviations

RT: Radiotherapy
RILT: Radiation-induced lung toxicity
RP: Radiation-induced pneumonitis
MLD: Mean lung dose
V20: Volume receiving more than 2000 cGy
SRP: Symptomatic radiation-induced pneumonitis
SARE: Severe acute radiation-induced esophagitis
FVC: Forced vital capacity
FEV1: Forced expiratory volume in the first second
PEF: Peak expiratory flow
DVH: Dose–volume histogram
RTOG: Radiation Therapy Oncology Group
CTCAE: Common Terminology Criteria for Adverse Events
ROC: Receiver operating characteristic
IMRT: Intensity-modulated radiation therapy
TOMO: Tomotherapy
3D-CRT: 3D-conformal RT
IL: Ipsilateral lung
TL: Total lung
MED: Mean esophageal dose
AUC: Areas under the curve
CT: Computed tomography
